# Psychometric Properties of Scared-C Scale in a Romanian Community Sample and Its Future Utility for Dental Practice

**DOI:** 10.3390/children9010034

**Published:** 2022-01-01

**Authors:** Sorana-Maria Bucur, Adela Moraru, Beata Adamovits, Eugen Silviu Bud, Cristian Doru Olteanu, Luminița Ligia Vaida

**Affiliations:** 1 Faculty of Medicine, Dimitrie Cantemir University, 540545 Târgu-Mureș, Romania; bucursoranamaria@gmail.com; 2 Faculty of Psychology, Dimitrie Cantemir University, 540545 Târgu-Mureș, Romania; moraruadela@gmail.com (A.M.); adamovitsbeata@gmail.com (B.A.); 3 Faculty of Dental Medicine, University of Medicine and Pharmacy, Science and Technology George Emil Palade, 540139 Târgu-Mureș, Romania; 4 Faculty of Dental Medicine, Iuliu Hațieganu University of Medicine and Pharmacy, 400012 Cluj-Napoca, Romania; 5 Department of Dentistry, Faculty of Medicine and Pharmacy, University of Oradea, 410087 Oradea, Romania; ligia_vaida@yahoo.com

**Keywords:** child anxiety disorders, screening, SCARED-C, psychometrics, dental practice

## Abstract

The psychological management of children and adolescents in need of pedodontics or orthodontic treatments continues to be an essential objective in dental activity because along with the accuracy of the techniques that are used, anxiety reduction, and knowledge of how to approach the patient are necessary for the treatment to be successful. Therefore, our study aimed to validate the Screen for Child Anxiety Related Emotional Disorders questionnaire, the child version of 41 items (SCARED-C) in the Romanian population for later use in pediatric dentistry. The instrument showed moderate to good internal consistency (α Cronbach from 0.63 to 0.91 for the total scale) and good test–retest reliability (0.70) on a subset of a sample comprising 85 children. A confirmatory factorial analysis (CFA) was conducted to test the factor structure of the Romanian version of the SCARED-C; the results showed that SCARED-C has good psychometric properties that can be used for screening anxiety in Romanian children and adolescents. The implications of using the SCARED-C in dental practice are discussed. Future studies need to be conducted to explore the convergent and discriminative validity of the instrument and its sensitivity to current DSM-V criteria. Application on a pediatric dental sample is also required.

## 1. Introduction

### 1.1. General Introduction to the Studied Problem

The psychological management of children and adolescents in need of pedodontics or orthodontic treatment continues to be an essential objective in dental activity because along with the accuracy of the techniques that are used, anxiety reduction and knowledge of how to approach the patient are necessary for the success of the treatment. Usually, the fear of pain or trauma is what causes anxiety. In turn, anxiety accentuates and amplifies the pain that is caused by the dental procedures that are performed, thus creating a vicious cycle that must be interrupted through intervention by the doctor [1].

The present paper is focused on validating the SCARED-C Scale for the Romanian population, and the motivation for choosing this psychological test is that it can determine the patient’s type of anxiety, which is crucial for the future approach of the dentist and overall collaboration with the patient. The dentist’s attitude and intervention to reduce the patient’s anxiety can be later adapted to the type of anxiety demonstrated by the patient who completed the questionnaire.

### 1.2. The Importance of the Problem–Arguments in Mental Health and Dental Practice. Definition of Terms

General anxiety is considered an irrational fear without a motivation known to the subject and that is unexplained by another mental illness or organic condition [2]. It is a permanent or almost permanent emotional condition that cannot be influenced by the patient himself/herself. Anxiety manifests itself either acutely when we are dealing with state anxiety or continuously and diffusely, in which case, we are dealing with general anxiety or anxiety as a trait [3].

Clinical anxiety can present with many forms that have been described by the fifth version of the Handbook of Diagnosis and Statistics of Mental Disorders, which was developed by the American Psychiatric Association (APA) [4].

Separation anxiety is one of the most common anxiety disorders seen in childhood, along with generalized anxiety and specific phobias. According to the American Psychiatric Association, the main feature of separation anxiety is the fear that is caused by the idea of separation from family, from people to whom the person has a strong emotional attachment with, or from home that is greater than natural concerns that are specific to the degree of a child’s mental development [4]. Separation anxiety is a normal psychological condition in early childhood [5] but presents a risk of influencing the child’s social and emotional functioning when it persists at older ages and can lead to the avoidance of certain places, activities, and experiences that are necessary for healthy development. Separation anxiety disorder with severe symptoms can lead to dropping out of school and disruptions to a child’s education [6]. Separation anxiety has been proven to be strongly correlated with dental anxiety and phobia [7].

In the same psychiatric classification, general anxiety is characterized by deep, unrealistic, and exaggerated concern about various situations that are perceived as threatening [4]. It is the exaggerated fear and worries about common things. These individuals find it difficult to control their anxiety. The presence of increased anxiety affects the mental development of children and their daily activities. A diagnosis of generalized anxiety is made if excessive care is present almost every day for 6 months and if the person has difficulty controlling anxiety. In addition, the person may experience one or more of the following symptoms: irritability, fatigue, insomnia, and concentration problems (does not feel able to think) [4]. Children and adolescents with general anxiety will have higher values of dental anxiety than other children [8,9].

Social anxiety is a common psychological disorder that causes other behavioral, psychological, even psychiatric problems such as severe anxiety and depression [10]. Young people with social anxiety could feel fear and may even experience difficulties in normal interactional situations, which have a negative impact on their social life [11]. Their fears are exacerbated in the presence of a factor that is considered to be potentially aggressive, such as dental treatment. Social anxiety was already included in the psychiatric disorders category in the third edition of the Diagnostic and Statistical Manual of Mental Disorders; since then, this condition has been regarded s as a phobia that is caused by various social situations [12]. Social phobia consists of the fear of evaluation and negative judgment from other people [13]. This is why people with social phobia are afraid to do things that could humiliate them in front of others. Those who suffer from social phobia may feel fear in a specific or in several situations, eventually leading to isolation [14].

The American Psychiatric Association describes another emotional disorder called selective mutism that often coincides with social phobia, which as comorbidity rates of up to 97% [15]. This psychological disorder could manifest in childhood or adolescence and is characterized by the inability to speak in specific social situations, such as in the classroom or in the dental office; however, in common situations, such as at home with the family, the individual is able to speak and communicate easily [15,16].

It has been demonstrated that a high level of dental anxiety (dental fear) has a strong positive correlation with a high level of comorbid phobias such as depression, mood disorders, and other psychiatric pathologies and symptoms. It is not possible to establish exactly whether patients with dental anxiety are more prone to having other psychiatric disorders or whether other psychiatric conditions are the primary disorders that predispose an individual to the development of dental anxiety [17]. The challenges facing dentists are how to deal with a patient with dental anxiety or how to choose the easiest method of treatment and to advise the patient to consult a psychologist or a psychiatrist [17].

### 1.3. Methods of Screening Children’s Anxiety in Psychological or Dental Practice

A psychological diagnosis is made by various methods, either by using test batteries or by clinical interviews. There is currently a wide range of methods and techniques that are available to study anxiety: one of the most used methods that provides reliable information is that of tests and questionnaires. Often, the tests do not replace the clinical diagnosis of a specialist but can help to highlight symptoms of anxiety that are indicated to be alleviated by various methods that can be implemented by dentists [18,19,20].

Very often during clinical activity, the dentist or the orthodontist might notice a patient’s anxiety or changes in their behavior. Even for experienced doctors, the approach to an anxious patient is often empirical and is based on outdated techniques or concepts. The doctor and the patient are two people facing each other from different positions, and both are in stressful situations. The communication between them becomes even more difficult when the doctor is at the beginning of his career, is a less empathetic person, or is stressed by the burden of professional responsibility, the difficulty of the case, or poor professional training [21]. These two people need to adjust to each other, something that is necessary for the doctor to control the situation and to reduce the patient’s anxiety by directly influencing the patient and by properly organizing his work and the environment of the dental office.

The use of questionnaires to assess anxiety could provide practical information for the dentist and may also bring psychological benefits to the patient. Dayley et al. (2002) demonstrated that by completing an anxiety assessment questionnaire during the first medical visit to a new health care professional, the anxiety experienced by anxious patients decreased; this may be due to the belief that the doctor may be treating them with increased care because they are aware of the fears that the patient already has [22]. A series of questionnaires that can be used to investigate dental anxiety have been developed and validated over the years [23]: The Childrens’ Fear Survey Schedule Dental Subscale (CFSS- DS), the Dental Fear Schedule Subscale (DFSS), the Dental Anxiety Scale (DAS), and the Modified DAS, Modified child dental anxiety scale (MCDAS), Facial Image Scale (FIS), Venham Picture Scale (VPS), Dental Fear Survey (DFS), Smiley Faces Program (SFP), and Revised SFP are only some of them.

### 1.4. SCARED and Its Proven Use

Having an anxiety screening tool such as the questionnaire designed by Birmaher et al. (1997) called the SCARED—Screen for Child Anxiety Related Disorders [24,25] helps to identify the symptoms of anxiety; this test is a quick, low-cost method that provides detailed information about the type anxiety that a patient is experiencing. In this study, we aimed to examine the psychometric data of this test and to validate the test’s use in the Romanian population for later use in dental practice. The doctor’s attitude and intervention to reduce the patient’s anxiety can be adapted to the type of anxiety being demonstrated by the patient who completed the questionnaire during later treatment.

The SCARED test has undergone various evaluations and changes over time, and the number of items has been reduced to 38; it has two versions, one for parents and one for children [25,26]. One scale asks parents questions about their child, and the other scale asks the child the same questions directly. These items evaluate five types of anxiety according to the DSM-IV classification [27]. There are five large subscales that are represented in the study: generalized anxiety, social anxiety, panic disorders, separation anxiety, and school avoidance. With the apparition of the DSM-V, school anxiety no longer appears among the types of anxiety, but the test remains useful because the other four sub-scales are valid and have adequate psychometric values [28]. When the test was initially published in 1997 [24], the test had average agreement between the parent–child assessment (correlation coefficients of 0.37–0.62), so we only analyzed and used the items addressed to children and adolescents with good internal consistency (a = 0.7–0.9) in this study, and the test–retest fidelity demonstrated high scores (*p* = 0.6–0.9) as well. The test was also analyzed with the Childhood Behavior Checklist (Achenbach and Edelbrock, 1983) [29] and the STAIC (Spielberger, 1973) [30], and the validity of the assessment was validated. In the final version comprising 41 items, Birmaher et al. (1999) [26] added 3 more items to assess social phobia. The test was designed for the English population and has been translated and validated on the Dutch (Hale et al., 2005) [31], German (Weitkamp et al., 2010) [32], Italian (Crocetti et al., 2009; Ogliari et al., 2006) [33,34], Belgian (Muris et al., 2002) [35], Spanish (Vigil-Colet et al., 2008) [36], Chinese (Su et al., 2008) [37], and South African (Muris et al., 2006) populations [38].The original instrument and other cross-cultural studies have demonstrated good internal consistency (α from 0.74 to 0.93) and optimal test–retest reliability (from 0.70 to 0.90).

The items on the SCARED test are short and simple sentences, and the subjects are asked to choose, based on their mood over the course of the last three months, whether the statement is "Untrue or rarely true", "Somewhat true or sometimes true", or "Very true or very often". These answers represent a score from 0 to 2. Each item represents one of the five subscales that the test was designed for. There are 13 items to check for panic symptoms (e.g., when you scare me, it’s hard for me to breathe), 9 items for generalized anxiety (e.g., I’m worried things won’t work out the way I want.), 8 items for separation anxiety (e.g., If I sleep somewhere other than at home, I feel scared), 7 items for social anxiety (I feel anxious when I’m with people I don’t know well.), and 4 items for school phobia (I have a stomach ache at school.) The higher the scores, the more severe the anxiety symptoms are. The test’s authors set a minimum score of 25 [25], above which an anxiety disorder can be assumed. Because this test is just an anxiety screening tool, the authors also recommend a clinical interview to make a final diagnosis.

Usually, anxious children are not observed properly in the general population or at school and home or in social settings because they are culturally considered to be “good kids”, as they tend to be compliant and submissive, and they do not create “problems” for adults to notice them. The SCARED-C is one of the best screening instruments for anxiety disorders according to DSM-IV criteria and that has cross-culturally good validity and reliability, as samples from the USA, Europe, Africa, and China have proved in a meta-analytical study [39]. 

The test is useful for children between the ages of 8 and 18 in the general and clinical population [31,38]. The biggest advantage is the free accessibility of the test and the fact that administration does not exceed more than 10 minutes.

The aim of our study was to validate the SCARED-C psychologic questionnaire (the child version, 41 items) in the Romanian population for later use in pediatric dentistry.

### 1.5. Study Hypotheses

To our knowledge, this is the first systematic study on the psychometric properties of the SCARED-C screening instrument in Romania. The following hypotheses were generated for the present study:

1. The mean and standard deviation norms in our Romanian sample would be similar to those that were obtained in other studies in different countries;
2. The factor structure of SCARED in the Romanian sample is similar to the original study of Birmaher et al. (1997) [24]. Confirmatory factor analysis will prove a better fit for the five-factor model of anxiety explained by the SCARED than it will for the one-factor model;
3. Girls have higher levels of anxiety than boys on the Romanian version of SCARED; by using the same instrument, early adolescents and middle adolescents demonstrate higher anxiety values than older adolescents;
4. The Romanian version of the SCARED has good internal consistency and test–retest reliability for further use in research and in clinical practice.

## 2. Material and Methods

### 2.1. Participants

The present study included 477 children and adolescents from different primary, junior high, and high schools from two public schools in Târgu Mureș, a town situated in the Central region of Romania. Informed consent was collected from the parents of the minors involved in the study. From the total sample, 51.5% were boys (244 children), and 48.9 were girls (210 children). Participants were children aged 8 to 19 years old (M = 14.2, SD = 2.8), and the participants were divided into three age groups: 56 early adolescents, aged 8–10 (11.7%), 160 middle adolescents, 11–14 years old (33.5%), and 261 older adolescents, 15–19 years old (54,7%). Most of the children were Romanian (74.8%), some were Hungarian (3.45%), and 21.8% did not declare their nationality. A total of 371 participants declared that they lived in an urban environment (77.8%), 57 indicated that they lived in a rural environment (11.9%), and 106 (22.2%) did not respond. From the total sample, only 84 children could be re-tested with the same instrument one month later to determine the test–retest reliability of the Romanian version of the SCARED-C scale.

### 2.2. Procedure

The pupils in the study group completed the SCARED-C (Screen for Child Anxiety Related Emotional Disorders) designed by Birmaher et al. [24]. Two of the authors had been trained in how to apply the questionnaire to children so that any influences in completing the answers by the participants were eliminated. Two public schools permitted the test to be applied, and some of the classes agreed with the informed consent. The confidentiality and anonymity for participation were assured. The tests were administered during classes with the participation of the teachers.

For the primary classes, a few additional comments and explanations were necessary, which were handled by two of the authors. The scale was completed by all participants individually, with group instruction for all. The duration for completion was about 20 minutes in total. Pupils who were absent on the day of testing were not assessed. All pupils received the following written instructions: “Below is a list of sentences that describe how people feel. Read each phrase and decide if it is ‘Not True or Hardly Ever True’, ‘Somewhat True or Sometimes True’, or ‘Very True or Often True’ for you. Then, for each sentence, fill in one circle that corresponds to the response that seems to describe you for the last 3 months. There are no right or wrong answers. Answer honestly about how you feel. Please do not skip any statements.” These instructions were printed above each questionnaire. After completion, the authors collected the scales, which were used for further.

### 2.3. Measure

The authors selected the SCARED-C (41-items) elaborated by Birmaher et al., [24].

The Romanian version of the SCARED test adaptation procedure consisted of several phases. First, after asking for permission from the authors, the SCARED-C screening instrument was translated into the Romanian language and then translated back into English by two different experts in both English and psychology. Both versions were compared, and inconsistencies were resolved. The translation aimed to find conceptual equivalents of words or phrases in Romanian and did not aim to translate the questionnaire word-for-word. Technical or overly scientific terms and academic language was avoided. Given that the test was also administered to adolescents, a common, colloquial language was used. The retroversion was completed by a translator who did not know the SCARED test, after which the version used in the present study was complete. During the second phase, the SCARED-C screening tool was used in this pilot study to determine the initial psychometric properties of the Romanian version.

### 2.4. Data Analysis

The demographics of the participants were analyzed using means, standard deviations, and frequencies using SPSS 19.0, IBM, New York, USA. Skewness and kurtosis indices were calculated to determine the normality of the variable’s distribution. Based Birmaher’s cut-off scores, the Romanian norms for anxiety and non-anxiety groups were explored through the use of two tests: the t-test and the MANOVA, in SPSS 19.0. The construct validity of SCARED-C was examined by using two types of Confirmatory Factorial Analysis (CFA): a one-factor model and a five-correlated factor model. The one-factor model comprised latent construct anxiety, while the five-factor model comprised the latent construct of the five types of anxiety disorders included in the theoretical model of the 41-item SCARED-C version: panic disorder/somatic symptoms, generalized anxiety disorder, separation anxiety, social anxiety, and significant school avoidance. CFA was calculated using Lavaan on a free website application called CBDI (Classical and Bayesian Development Instrument–Beta Version) that was developed by Karanevich et al. (2015) [40]. Because the SCARED-C is rated on a three-point Likert scale, we used a diagonally weighted least squares (DWLS) estimator rather than using the maximum likelihood (ML) estimator in the CFA. The following cutoffs in different fit indices were used to determine an acceptable model: normed chi-square (i.e., chi-square value divided by the degrees of freedom) < 3, the comparative fit index (CFI) > 0.85 and Tucker–Lewis index (TLI) > 0.90, root mean square of error approximation (RMSEA), and standardized root mean square residual (SRMR) < 0.08. The model that had the indices with the best fit was then explored for sex and age differences. To examine the reliability and internal consistency of the scale, the Alpha Cronbach coefficient was calculated as well a s the Pearson correlation coefficients to determine test–retest administration using SPSS 19.0.

## 3. Results

### 3.1. The Romanian SCARED Norms (Hypothesis 1)

Descriptive statistics were conducted to explore the normality of distribution. Table 1 presents the means, standard deviations, and skewness and kurtosis indices for the overall scores and subscales of the Romanian version of the SCARED-C. The results showed an asymmetrical distribution, with skewness and kurtosis indices that were higher than the critical value 1 for the total score and the scores for the two subscales (Panic Disorder PD and School Anxiety SA). One reason for this asymmetry could be the existence of two sub-groups in our sample: anxious and non-anxious children and adolescents. This hypothesis was explored further in Table 2, where the descriptive and t-test of mean differences are presented.

Birmaher et al. (1997) specified a cut-off point of 25 for identifying clinical anxiety scores on the SCARED (overall score). In our sample of 477 children and adolescents, we could identify 355 people (74.4%) with normal anxiety and 122 children (25.6%) with clinical anxiety based on cut-off score of 25 according to the SCARED screening instrument. Table 2 shows significant discrepancies between the mean values of the anxious and non-anxious groups and the t-values on the overall score and its subscales. These differences are statistically significant at *p* < 0.01 for both the overall score and for the scores of the SCARED subscales; school avoidance (SA) was the only subscale that was significant, at *p* < 0.05. We can conclude that a) the initial asymmetry of the distribution is due to these sub-groups and that b) the Romanian version of the SCARED is a sensitive instrument that can discriminate between anxious and non-anxious children, even in a community sample (normal population). 

The means and standard deviations that were obtained in the Romanian sample are quite close to the values that were identified by Birmaher et al. (1999) [26] in both anxious and non-anxious groups. Similar results were obtained in Italian samples (Crocetti et al., 2009; Ogliari et al., 2006) [33,34] and in Dutch samples (Hale et al., 2005) [31]. Overall, as proven by the meta-analytical study conducted by Hale et al., 2011 [39], there is good support for using this instrument cross-culturally in mental health practices. Our study fills the gap of missing Romanian norms by showing similar values with other European samples. Hypothesis 1 is confirmed.

### 3.2. The Romanian SCARED Structure (Hypothesis 2)

The factor structure of the Romanian version of the SCARED-C was examined by means of Confirmatory Factor Analysis (CFA). As it can be seen from Table 3, the one-factor model has a bad fit (chi-square normed higher than 3), the CFI, TLI, and RMSEA indices show an acceptable fit, and SRMR indicates a bad fit for the overall sample. 

The five-factor model had a significantly better fit than the one-factor model, as shown by the chi-square (a value of 1.90, less than critical value of 3, *p* < 0.01). The five-factor model of the overall sample corresponds to the theoretical model proposed by Birmaher et al. (1997) [24] and includes the following factors: panic disorder (PD)/somatic symptoms (SS); generalized anxiety disorder (GAD); separation anxiety (SAD); social anxiety (SC); and school avoidance (SA). All model fit indices critical values ranging from acceptable fit (SRMR) to good fit (CFI, TLI, RMSEA) and to very good fit (normed chi-square). Hypothesis 2 is confirmed.

The factor loadings and Pearson item–total correlation are presented in Table 4. Factor 1 (PD) include 13 items, with values ranging from 0.40 for Item 19 “I get shaky” to the highest level of 0.88 for Item 6 “When I get frightened, I feel like passing out”. The item–total correlation values for this factor are medium, ranging from 0.36 for Item 34 to the highest level of 0.56 for Item 24. 

Factor 2 (GAD) is loaded with nine items, with the lowest factor-loading value being 0.54 for “I worry about other people liking me” (Item 5) to the highest level of 0.76 for Item 23—“I am a worrier.” The item–total correlations for this factor range from 0.39 to 0.52.

Factor 3 (SAD) has eight items, with saturation values ranging from 0.42 for Item 22 “I don’t like to be away from my family.” To a value of 0.72 (Item 16) “I have nightmares about something bad happening to my parents.”. The item–total correlations range from 0.29 to 0.47 for the same items. 

Factor 4 (SC) has seven items, with factor loadings ranging from 0.41 (Item 3) “I don’t like to be with people I don’t know well.” to the highest value of 0.75 (Item 40) “I feel nervous when I am going to parties, dances, or any place where there will be people that I don’t know well.” The item–total correlations for these items are between 0.24–0.50.

Factor 5 (SA) has four items with factor loadings varying from 0.53 (Item 2) “I get headaches when I am at school.” to 0.78 (Item 36) “I am scared to go to school.” The Pearson item–total correlations for this factor vary between 0.27 and 0.39.

### 3.3. Sex and Age Difference within the Romanian Sample (Hypothesis 3)

The MANOVA results indicate that, when using the Wilks–Lambda criteria, the correlated dependent variables of the SCARED-C (anxiety subscales) are significantly influenced by sex (F(454) = 12.2, *p* < 0.001, η^2^ = 0.12) and age (F(474) = 15.2, *p* < 0.001, η^2^ = 0.13) but are not significantly influenced by their interaction (sex and age): F(453) = 0.88, *p* = 0.54, η^2^ = 0.01.

#### 3.3.1. Sex Differences

Girls were found to score significantly higher than boys in terms of the overall score (F(454) = 34.8, *p* < 0.001, η^2^ = 0.07) and on the panic disorder (F(454) = 20.6, *p* < 0.001, η^2^ = 0.04), generalized anxiety disorder (F(454) = 52.1, *p* < 0.001, η^2^ = 0.10), social anxiety disorder (F(454) = 20.5, *p* < 0.001, η^2^ = 0.04), and separation anxiety (F(454) = 11.0, *p* < 0.001, η^2^ =0.02) subscales. No significant differences were found for school avoidance (F(454) = 0.29, *p* > 0.05, η^2^ = 0.00). The results are shown below in Table 5.

#### 3.3.2. Age Differences 

It seems that anxiety decreases with age in our group. The MANOVA results and the Dunnett T3 post hoc tests showed that the first subgroup (8–10 years) scores were significantly higher than those of the older group (15–19 years) but that they were not significantly higher than those that were obtained for the middle group (11–14 years) in terms of the overall score (F(472) = 17.5, *p* < 0.001, η^2^ = 0.06) and for the panic disorder (F(454) = 20.6, *p* < 0.001, η^2^ = 0.04) social anxiety disorder (F(472) = 10.4, *p* < 0.001, η^2^ = 0.04) subscales. Age does not have a relevant impact on generalized anxiety disorder or school avoidance (Table 5). 

The results show higher anxiety values for girls than boys, and a decrease in anxiety with age; thus, Hypothesis 3 is confirmed.

### 3.4. Internal Consistency and Test–Retest Teliability of Romanian SCARED Version (Hypothesis 4)

The internal consistency of the Cronbach’s alpha coefficients were calculated for the overall score and distinct subscales. For the overall score, α was 0.91; 0.83 for the panic/somatic anxiety (PN) subscale; 0.81 for generalized anxiety (GD); 0.70 for separation anxiety (SP); 0.78 for social anxiety disorder (SC); and 0.63 for school avoidance (SH). At the time of retesting (*n* = 88), the internal consistency was 0.90 for the total scale; 0.81 for PN and GD; 0.66 for SP; 0.83 for SC; and 0.67 for SH. Both administrations of the Romanian version of the SCARED demonstrated very good internal consistency. The results that were obtained are consistent with the results of Birmaher et al. (1997).

The consistency of the Romanian version of SCARED over time was explored using test–retest reliability for the overall general scores and the subscales based on two evaluations of the same instrument at a one-month interval. The test–retest correlations achieved values between 0.47 and 0.70 (Table 6). The test–retest reliability in our Romanian sample was quite modest because it was difficult to find the same children and adolescents for retesting in schools after one month. Future studies can fix this problem with a more accurate design. The smallest coefficients were for separation anxiety (0.53), which decreased in adolescence and for school avoidance (0.47), an issue that was already discussed as having a weaker contribution to the total score of the SCARED. The results are presented in Table 6. Social anxiety (0.70) and the total scores (0.70) were the highest in consistency over time but were still at the lower limit of a good consistency measure. The Romanian version of the SCARED showed good consistency and test–retest reliability, proving to be a useful tool for research and practice, thus confirming our fourth hypothesis.

## 4. Discussion

The SCARED anxiety questionnaire has proven to be a tool with a high level of fidelity and validity in assessing anxiety symptoms in the Romanian population aged between 8 and 18. By using it, we can prevent the occurrence of anxiety disorders that can lead to other disorders that disrupt the daily life of children and that may cause behavioral problems in difficult situations, such as when seeing the dentist. Through these anxiety assessments and studies, mental health programs can be developed to educate both children and their parents on how to cope with stressful situations that cause anxiety, monitor symptoms, and learn coping methods to have a healthy and balanced life.

The test results suggest that in the case of children aged between 8–10, separation anxiety has the highest values in the studied group. Aminabadi et al. [41] indicated that a child’s behavior when confronting dental anxiety is positively correlated to the mother’s emotional intelligence; an authoritarian parenting style was predominantly found in mothers with high levels of emotional intelligence. They found significant correlations between an authoritarian parenting style and separation anxiety. The mother’s emotional intelligence may influence the child’s ability to cope with stressful conditions such as seeing the dentist. This means that by influencing a parent’s authoritarian parenting style, we may reduce separation anxiety.

A conclusion that can be drawn would be that, in the case of a patient with a high score on the elements of the SCARED test that are related to separation anxiety, the dentist should allow the mother to enter the office together with the child; the mother could hold the child’s left hand during the dental intervention or could remain to the left or in front of the little patient, allowing the mother to maintain eye contact that is meant sure encourage the child and to ensure a sense of safety. At the same time, we advise that the mother not to act with an excess of authority so that the child behaves appropriately; this is the responsibility of the medical team, in which the most important role is played by the doctor.

At this age, children are still dependent on parental approval, which they are almost always trying to achieve, avoiding punishments or quarrels in cases of failure or disobedience. Therefore, the influence of the parents and especially of the mother is an important factor for the success of the pedodontic or orthodontic treatment.

Our study shows that social anxiety is higher in adolescents. The fear of negative evaluation from others and self-focused attention maintain social anxiety. The social integration of adolescents depends on the projection of the self-image and his self-esteem. It has been recognized that the morphology of the dentofacial region significantly contributes to the overall appearance of the face [42]. This is where orthodontics plays a role in improving facial appearance. Children with a normal dental arrangement are perceived as being more handsome, smarter, and desirable as friends, and they may even be perceived has being superior mental qualities and being less prone to aggressive behavior by others of their own age and by adults [42]. On the other hand, de Jong showed the role of a negative self-image and low self-esteem in social anxiety [43]. People with social anxiety are also characterized by a difference between the perception of themselves and of others, and this tendency to discredit themselves is a main element of social anxiety. In these situations, the doctor will tactfully try to communicate better with the patient, creating a relationship based on trust and friendship. They may also feel vulnerable after traumatic dental experiences during childhood. Communication is facilitated by the fact that teenagers see dentists as professionally and materially independent people and this impresses them in a positive way.

Particular situations are children with selective mutism who are very difficult patients; working with them is extremely hard to achieve and requires several accommodation sessions with the dental practice environment, with the dentist, and with the support staff. The reason for this pathology is complex and controversial. It is unclear whether children with selective mutism cannot not speak because of overwhelming anxiety or whether withholding speech functions is an avoidance mechanism. Psychophysiological measures have indicated that children with selective mutism experience less arousal than other children during social interaction tasks; as such, the lack of speech may be an avoidance mechanism [44].

Selective mutism can have a complex etiology that may involve genetic, environmental, temperament, neurodevelopmental factors, and hereditary disorders [45,46]. Treatment may include cognitive, behavioral therapy, or pharmacotherapy. Commonly, dentists are not trained to deal with this type of patient, even if knowledge about the diagnosis and intervention techniques in these cases is crucial. For children with selective mutism, dental visits could be a challenge [45]. Each case is unique because the causes of behavioral deviance are variable. A child who needs dental emergency, regular treatment, or routine examination may not yet be diagnosed with selective mutism, so further knowledge of the condition using the SCARED-C scale could make it a tool that can be used for a presumptive diagnosis and for choosing the correct intervention to make collaboration possible. The dental team should be aware of the possible therapy the child has already received and work with these principles during dental programs. Knowing if the child has other phobias or anxiety symptoms can help; simple strategies such asking the parent how to communicate with the little patient or understanding what makes the child feel at ease can be very helpful to establish an approach [45].

In our study, girls scored significantly higher than boys in terms of the overall SCARED-C score. The girls scored significantly higher on the panic disorder, generalized anxiety, social anxiety, and separation anxiety subscales. No significant differences between the sexes were found for school avoidance. Our results are similar to those found in the literature. For example, Fredrikson et al. [46] found a higher incidence of specific phobias in women than in men. Compared to men, women indicated higher fear ratings for all objects and situations. Fear of injections decreased as a function of age in women but not in men [47]. McLean et al. [11] found that anxiety disorders are more prevalent and more disabling in women than they are in men. Women had higher rates of diagnosis for each of the anxiety disorders that were examined (generalized anxiety disorder, panic disorder, specific phobia, and post-traumatic stress disorder), with the exception of social anxiety disorder, which showed no gender difference in terms of prevalence. Halasa S. et al. [48] found significant differences between males and females in social anxiety and post-traumatic stress disorder, which were higher in females.

In our study group, anxiety was shown to decrease with age. The children between the ages of 8 and 10 years old scored significantly higher than the older group of teenagers who were 15–19 years of age but not compared to the middle group of youths who were 11–14 years of age on the overall score and on the panic disorder and social anxiety disorder subscales. There was no relevant impact of age on generalized anxiety disorder or school avoidance. Arab et al. [49] demonstrated the utility of using the SCARED in a study group of Arabian children and adolescents who were similar in age and found out that boys demonstrated a reduction in anxiety symptoms with age and that girls showed stable and elevated anxiety symptoms at any investigated age group, suggesting the need for intervention at an early age to prevent later mental health problems. Patients with elevated values of general anxiety showed higher increases in anxiety as a state, blood pressure, and peripheral pulse in the dental office under stress [50]. Anxiety in children and adolescents could be treated through the use of breathing control techniques and the Jacobson progressive muscle relaxation technique; both of these techniques have been proven to be effective in reducing anxiety as a condition and as a trait. Jacobson’s progressive muscle relaxation technique is more effective in reducing the variables that define anxiety than the breathing control technique is [50].

Several studies clearly indicate that anxiety and stressful situations are significantly involved in the initiation and maintenance of temporomandibular disorders (TMDs) [51,52,53,54,55,56]. These are important in the etiology of this complicated group of diseases [51,52,53,54,55]. TMDs are a multifactorial condition that could be associated with Class II and Class III malocclusions, where myofascial pain has a higher prevalence and where psychological factors, such as anxiety, are more prevalent [52,53]. TMD-related pain is more common in girls than in boys and concerns neuropsychological factors, as women appear to be more vulnerable to stress compared to men [51,54]. As such, anxiety plays an important role in the pathogenesis of TMD.

The risk of bruxism also increases in general anxiety cases and during particularly stressful periods. TMD and bruxism are frequently associated with orofacial pain, reductions in articular mobility, and even pain in physiologic mandibular movements [55,56,57]. Bruxism is frequently associated with the presence of non-carious cervical lesions that may cause hypersensitivity [58], which increases the patient’s stress and affects the patient’s quality of life.

There are other diseases that are positively correlated with anxiety, such as recurrent aphthous stomatitis, oral lichen planus, and burning mouth syndrome [59,60,61,62]. Anxiety is closely associated with the incidence of trigeminal neuralgia essential (TN) [63,64]. General anxiety is positively correlated with dental fear and the perception of dental pain [65].

Future orthodontic patients reported higher levels of orthodontic dental anxiety than current patients. Anxiety and bad habits are associated with malocclusions [66,67]. The fear of trauma (dental trauma, bone trauma) also causes anxiety [68].

The co-presence of anxiety, regardless of whether it is associated with mental problems or not, causes neglect, so even small issues that are related to poor oral hygiene can become serious, with the possible worsening of the periodontal situation in orthodontic patients and an increase in therapy times due to lack of cooperation [69,70]. Today, telemedicine and web channels are increasingly being used by patients to obtain health-related information [71] such as brushing techniques, oral hygiene maintenance, and pathologies such as mouth sores and oral thrush [72]. Virtual reality (VR) environments provide support to reduce the anxiety and the stress related to dental care and improve compliance with dentists. VR allows the patient to relax and be distracted by giving the feeling of a more pleasant location without stressful elements. VR may provide a possible alternative treatment for dental anxiety and phobia [73,74].

A clinical protocol for approaching anxious patients by using computer software based on anxiety values determined with the SCARED-C questionnaire could be a useful working tool that could be integrated into pediatric dentistry offices [75].

After validation by the present study, we intend to use this questionnaire in our practical activities to reduce anxiety and to collaborate with the patient according to the determined anxiety type. This will be the subject of another study that will describe changes in anxiety after psychological intervention.

The strength of our study is, as far as we know, that it is the first validation of the SCARED-C Scale for the Romanian population. The present study contributes to the increase in reference data in the literature, supporting future research in this field. The confirmatory analysis and the internal consistency values of the questionnaire provide valuable data for the validity of the SCARED-C in our country.

The limitations of our study lie in the small number of subjects who could be retested due to the pandemic conditions that restricted student access to schools because of the high COVID-19 infection rate. It would have been better and we could have obtained more accurate results if our study sample had been bigger. There is a great need to have and use psychological tests in our population; The practice of psychology was not introduced to our country until three decades ago due to the communist regime. There is still a lack of psychological tests that have been validated for the Romanian population. Our study has succeeded in offering a helpful tool for psychological investigation to all of the specialists who work in humanitarian fields and in medicine.

## 5. Conclusions

The Romanian version of the SCARED-C demonstrated good internal consistency and proved to be a sensitive psychometric instrument to discriminate between anxious and non-anxious children. Girls scored significantly higher than boys on the overall score, on panic disorder, generalized anxiety, social anxiety, and separation anxiety. No significant differences between the sexes were found regarding school avoidance. The overall anxiety score decreased with age, but age had no relevant impact on generalized anxiety or school avoidance. Dentists could use this questionnaire to determine the type of anxiety being experienced by minor patients before any intervention and may adapt their attitude or perform specific psychological interventions to make dental treatment possible.

## Figures and Tables

**Table 1:** Demographic means, standard deviations, and skewness and kurtosis of the anxiety subscales.

SCARED	Mean	Standard Deviation	Skewness	Kurtosis
Total score (TS)	19.1	12.2	1.0	1.1
Panic Disorder (PD)	4.4	4.1	1.8	4.0
Generalized Anxiety Disorder (GAD)	4.8	3.7	0.8	0.1
Separation Anxiety (SAD)	3.5	2.8	0.9	0.7
Social Anxiety (SC)	4.7	3.3	0.4	−0.5
School Avoidance (SA)	1.6	1.7	1.3	1.7

**Table 2:** Means, standard deviations, and t-tests for anxious and non-anxious children according to Romanian version of SCARED-C.

SCARED	Mean		Standard Deviation		T-Test	*p*-Value
	Non-Anxious Group	Anxious Group	Non-Anxious Group	Anxious Group		
Total score (TS)	13.3	35.7	6.5	9.1	−24.9	0.00
Panic disorder (PD)	2.7	9.0	2.0	4.9	−13.5	0.00
Generalized anxiety disorder (GAD)	3.2	9.4	2.4	3.1	−20.0	0.00
Separation Anxiety (SAD)	2.5	6.4	1.9	2.9	−13.7	0.00
Social Anxiety (SC)	3.6	7.9	2.7	2.8	−14.7	0.00
School Avoidance (SA)	1.2	2.8	1.4	1.9	−8.6	0.03

**Table 3:** Model fit indices for the one-factor model and the five-factor models of the Romanian version of the SCARED *(n* = 477).

Factor Structure Models	*n*	Chi-Square	CFI	TLI	RMSEA	SRMR
One-factor model Total sample	477	3.41 *p* < 0.01	0.930	0.926	0.071 *p* = 0.00	0.104
Five-factor model						
Total sample	477	1.90 *p* < 0.001	0.974	0.972	0.044 *p* = 0.99	0.082
Boys	267	1.44 *p* < 0.001	0.972	0.970	0.041 *p* = 0.99	0.104
Girls	133	1.42 *p* < 0.001	0.976	0.975	0.045 *p* = 0.90	0.099
Early adolescents	56	1.23 *p* < 0.001	0.967	0.965	0.065 *p* = 0.05	0.171
Middle adolescents	160	1.09 *p* < 0.05	0.994	0.993	0.024 *p* = 1.00	0.103
Older adolescents	261	1.44 *p* < 0.001	0.975	0.973	0.041 *p* = 0.997	0.106

Note: *n*: number of participants; CFI: comparative fit index; TLI: Tucker–Lewis Index; RMSEA: root mean square residual; SRMR: standardized root mean square.

**Table 4:** Factor loadings and factor internal consistency *(n* = 477).

SCARED Item	Panic Disorder	Generali–Zed Anxiety Disorder	Separation Anxiety	Social Anxiety	School Avoidance	Pearson Item–Total Correlation
S1 When I feel frightened, it is hard to breathe	0.62					0.44
S6 When I get frightened, I feel like passing out.	0.88					0.46
S9 People tell me that I look nervous.	0.60					0.41
S12 When I get frightened, I feel like I am going crazy.	0.73					0.52
S15 When I get frightened, I feel like things are not real.	0.60					0.42
S18 When I get frightened, my heart beats fast.	0.40					0.31
S19 I get shaky.	0.67					0.52
S22 When I get frightened, I sweat a lot.	0.66					0.47
S24 I get really frightened for no reason at all.	0.81					0.56
S27 When I get frightened, I feel like I am choking.	0.84					0.49
S30 I am afraid of having anxiety (or panic) attacks.	0.75					0.51
S34 When I get frightened, I feel like throwing up.	0.73					0.36
S38 When I get frightened, I feel dizzy.	0.79					0.51
S5 I worry about other people liking me.		0.54				0.39
S7 I am nervous.		0.58				0.43
S14 I worry about being as good as other kids.		0.68				0.51
S21 I worry about things working out for me.		0.65				0.50
S23 I am a worrier.		0.76				0.54
S28 People tell me that I worry too much.		0.68				0.48
S33 I worry about what is going to happen in the future.		0.70				0.52
S35 I worry about how well I do things.		0.67				0.49
S37 I worry about things that have already happened.		0.69				0.52
S4 I get scared if I sleep away from home.			0.64			0.40
S8 I follow my mother or father wherever they go.			0.59			0.38
S13 I worry about sleeping alone.			0.71			0.39
S16 I have nightmares about something bad happening to my parents.			0.72			0.47
S20 I have nightmares about something bad happening to me.			0.61			0.43
S25 I am afraid to be alone in the house.			0.70			0.38
S29 I don’t like to be away from my family.			0.42			0.29
S31 I worry that something bad might happen to my parents.			0.55			0.38
S3 I don’t like to be with people I don’t know well.				0.41		0.24
S10 I feel nervous with people I don’t know well.				0.68		0.44
S26 It is hard for me to talk to people I don’t know well.				0.72		0.47
S32 I feel shy with people I don’t know well.				0.74		0.47
S39 I feel nervous when I am with other children or adults and I have to do something while they watch me (for example: read aloud, speak, play a game, play a sport).				0.70		0.47
S40 I feel nervous when I am going to parties, dances, or any place where there will be people that I don’t know well.				0.75		0.50
S41 I am shy.				0.71		0.45
S2 I get headaches when I am at school.					0.53	0.27
S11 I get stomachaches at school.					0.76	0.39
S17 I worry about going to school.					0.74	0.36
S36 I am scared to go to school.					0.78	0.30

**Table 5:** Gender and age differences and effect size (MANOVA results).

SCARED	Gender			Effect Size	Age				Effect Size
	Boys (*n* = 244)	Girls (*n* = 210)	F	Partial Eta Square	8–10	11–14	15–19	F	Partial Eta Square
Total score	16.0 ± 10.91	22.52 ± 12.66	34.8	0.07 Medium	24.78 ± 13.30	21.66 ± 13.35	16.31 ± 10.35	17.5	0.07 medium
Panic Disorder/Somatic Symptoms	3.59 ± 3.50	5.33 ± 4.67	20.6	0.04 small	6.33 ± 5.27	5.29 ± 4.56	3.44 ± 3.22	18.1	0.05 Medium
Generalized Anxiety Disorder	3.69 ± 3.20	6.12 ± 3.59	52.1	0.10 Medium	5.33 ± 4.01	4.93 ± 4.00	4.67 ± 3.57	0.78	0.00 Small
Separation Anxiety	3.07 ± 2.64	3.94 ± 2.89	11.0	0.02 small	5.92 ± 3.15	4.33 ± 2.90	2.49 ± 2.12	54.3	0.18 Large
Social Anxiety	4.05 ± 3.24	5,45 ± 3.31	20.5	0.04 small	5.23 ± 2.80	5.55 ± 3.57	4.09 ± 3.21	10.4	0.04 Medium
School Avoidance	1.58 ± 1.88	1.67 ± 1.66	0.29	0.00 small	1.94 ± 1.93	1.54 ± 1.66	1.60 ± 1.73	1.1	0.00 Small

**Table 6:** Alpha Cronbach total and subscales and test–retest reliability.

SCARED	Alpha Cronbach Coefficient Test 1 (*n* = 477)	Alpha Cronbach Coefficient Test 2 (*n* = 88)	Test 1 (m ± sd)	Test 2 (m ± sd)	*r*_12_
Total score (TS)	0.91	0.90	19.10 ± 12.20	14.77 ± 10.39	0.70 **
Panic Disorder (PN)	0.83	0.81	4.40 ± 4.13	3.23 ± 3.50	0.63 **
Generalized Anxiety (GD)	0.81	0.81	4.84 ± 3.77	3.70 ± 3.37	0.64 **
Separation Anxiety (SP)	0.70	0.66	3.51 ± 2.81	2.08 ± 2.10	0.53 **
Social Anxiety (SC)	0.78	0.78	4.72 ± 3.36	3.94 ± 3.13	0.70 **
School Avoidance (SH)	0.63	0.63	1.62 ± 1.73	1.65 ± 1.77	0.47 **

Note: ** Value is significant at p < 0.01

## Data Availability

Data supporting reported results can be found by contacting Adela Moraru, moraruadela@gmail.com.
